# Development of a novel GP continuity measurement for practices without personal lists: a two-practice pilot of the modified SLICC

**DOI:** 10.3399/BJGP.2025.0624

**Published:** 2026-08-25

**Authors:** Kate Sidaway-Lee, Denis Pereira Gray, Jason Fearn-Smith, Alastair Buick, Serge Engamba, Nada Khan, Philip Hugh Evans

**Affiliations:** 1 St Leonard’s Research Practice, Exeter, UK; 2 University of Exeter Medical School, Exeter, UK; 3 Southernhay House Surgery, Exeter, UK; 4 Exeter City Primary Care Network, Exeter, UK; 5 St Leonard's Practice, Exeter, UK; 6 Exeter Collaboration for Academic Primary Care (APEx), University of Exeter, Exeter, UK

**Keywords:** continuity of care, general practice, measurement, primary care

## Abstract

**Background:**

It is widely recognised that continuity of GP care should be improved. This requires a continuity measure that is applicable to all practices. Previous measures have limitations.

**Aim:**

To develop an improved measure of continuity of care and compare its performance with established measures.

**Design and setting:**

Audit data were used to conduct a 1-year pilot of the measure in two suburban general practices in Devon, England.

**Method:**

The modified St Leonard’s Index of Continuity of Care (mSLICC) was developed; scores were calculated monthly, and compared with those generated using the St Leonard’s Index of Continuity of Care (SLICC), the 1-year Bice–Boxerman Continuity of Care (BB), and Usual Provider of Care (UPC) indices. Proportions of patients and appointments included were calculated, with patients grouped by age, sex, 3-year appointment numbers, and frailty categories.

**Results:**

In the two practices, the mSLICC included 19 840 of 29 127 (68.1%) appointments and a mean of 65.2% (standard deviation [SD] 6.4%) of patients with appointments. The UPC and BB included 21 032 (72.2%) appointments and 4096 (41.3%) of 9924 patients with appointments. In practice A, the mSLICC score was 52.0%, the SLICC score was 57.1%, the mean UPC score was 0.63 (95% confidence interval [CI] = 0.62 to 0.64), and the mean BB score was 0.41 (95% CI = 0.40 to 0.43). In practice B, the mSLICC score was 25.9%, the SLICC score was 25.3%, the mean UPC score was 0.50 (95% CI = 0.49 to 0.51), and the mean BB score was 0.23 (95% CI = 0.22 to 0.25). The mSLICC correlated with the SLICC (*r* = 0.98, *P*<0.001).

**Conclusion:**

The mSLICC is a new monthly measure of continuity. It has fewer limitations than the BB and UPC, and does not require a named GP. With sufficient IT resources, the mSLICC could be used to measure continuity of care in practices that do not use personal lists.

## How this fits in

Continuity of care is associated with better outcomes; however, in many UK practices, GPs do not hold personal lists, making some existing measures difficult to apply. Commonly used research measures — such as the Usual Provider of Care Index and the Bice–Boxerman Continuity of Care Index — are unsuitable for rapid feedback and quality improvement because they require a certain length of time, exclude many patients, and give less weight to the appointments of frequent attenders. The modified St Leonard’s Index of Continuity of Care (mSLICC) is a new, practical measure that works in all practices — including those without personal lists — and can be calculated monthly, allowing closer monitoring of continuity levels, although 2 years’ prior data are required to identify a regular GP. By including more patients and avoiding key biases, the mSLICC could enable practices and policymakers to monitor and improve continuity in real time.

## Introduction

Relational continuity of care, characterised by a continuing doctor–patient working relationship, is associated with a range of benefits for patients, doctors, and health systems.^1–5^ The vast majority of research has studied doctors and their patients, not other primary care professionals.

There is growing interest in improving relational continuity in general practice, given its association with beneficial patient outcomes, but there are difficulties in agreeing the optimal way to measure continuity across all general practices. There are key features that an effective measure requires; these are outlined in Box 1.

Box 1. **Key features of effective measures of continuity of care**

For quality-improvement projects and for monitoring provision of continuity, a measure should:

- give meaningful results within a reasonable timetable so it rapidly reveals any changes in continuity levels;
- include most, and ideally all, patients who had appointments, and most appointments provided, ideally all;
- be statistically reliable — have face validity so that GPs understand whether they are providing better continuity or not; and
- capture meaningful continuity in all practices.

The three most common methods of measuring continuity — namely, the St Leonard’s Index of Continuity of Care (SLICC),^6–8^ the Usual Provider of Care Index (UPC),^9^ and the Bice–Boxerman Continuity of Care Index (BB)^10^ — were developed in 1974, 1975, and 1977 respectively, but their different statistical and other properties have varying clinical implications.

A minority of practices have a working personal list system,^11^ which means that each patient has a named GP and can expect to see this GP for a reasonable proportion of their GP appointments. In these practices, the SLICC^4^ can be measured both monthly and for individual GP lists, and includes all GP appointments. It is particularly good for health-improvement projects, enabling rapid evaluation of the effect of an intervention on continuity. However, many practices, despite officially having a named GP for all patients, do not encourage patients to consult with that GP;^12^ for those practices without personal lists, the SLICC may not effectively capture the continuity provided. The UPC and BB do not require there to be a previously designated GP, but there are multiple problems with using these measures to monitor continuity improvement in practices^13^ as they were designed for research. These problems make these measures less useful for health-improvement projects that require rapid, clear feedback to facilitate change.

A measure is needed that has the advantages of the SLICC, in terms of monthly measurement and including less-frequent attenders, while eliminating the requirement for a named GP with whom the patient should expect continuity. This study aimed, therefore, to develop, pilot, and assess the feasibility of a novel measure — namely, the modified St Leonard’s Index of Continuity of Care (mSLICC) — and to compare it with the measures that are most commonly used in research and proposed^14^ as continuity metrics for general practices.

## Method

### Setting and data extraction

This study used GP appointment data from April 2022 until March 2025 that was extracted from two suburban practices (practice A and practice B) in Devon, England, using a SystmOne clinical system for a primary care network (PCN) continuity of care audit. Both practices have Index of Multiple Deprivation 2019^15^ scores that indicate slightly less deprivation than the English average.^16^ Practice A had ∼9500 patients, with seven to eight list-holding GPs, a sessional GP, a GP registrar, and locums reviewing patients during the study period. The practice has a strong continuity focus: most routine appointments are booked with the named GP, whereas on-the-day appointments are with the duty doctor. During the study period, some of the GPs had unexpected and prolonged absences from the practice.

Practice B had ∼8000 patients with four to five list-holding GPs, a sessional GP, a GP registrar, and locums reviewing patients during the study period. The practice values continuity and encourages patients to book with their preferred GP. Urgent same-day appointments were with available or duty doctors. During the study period, one full-time partner left and was primarily covered by long-term locums.

### Appointment inclusion and exclusion

All telephone and face-to-face appointments with an identifiable doctor were included, including those with patients and GPs who had left and joined during the year and the previous 2 years. Appointments without a specific GP (for example, those with unnamed doctors, such as ‘Dr Triage’ or similar) were excluded. As far as possible, ‘appointments’ that were administrative tasks (flagged with ‘patient not present’ or ‘online/written’) were also excluded.

### Continuity measures

SLICC and mSLICC scores were calculated monthly from April 2024 until March 2025, the 2 prior years’ data being used to identify a regular GP for the mSLICC. The SLICC gives the percentage of GP appointments with the named, personal GP;^7^ named GPs were as on 31 March 2025. The mSLICC gives the percentage of all GP appointments that were with ‘regular’ GPs, with ‘regular’ defined as the most-seen GP, with at least two appointments with the patient within the 2 years prior to each separate month for which continuity was being measured. If two GPs had an equal number of appointments, the GP who saw the patient most recently was chosen. Some patients had no regular GP and, therefore, were excluded from the mSLICC (Supplementary Figures S1 and S2).

In addition, BB and UPC scores (Supplementary Table S1) were calculated as previously described,^13^ although patients were excluded from these measurements if they had fewer than three appointments in the year of measurement. This timeframe and cut-off were chosen to optimise statistical reliability while having a timescale that meant that any change in continuity might be detected. UPC and BB scores were calculated at a patient level, then presented as a mean of the individual scores. These were calculated as a monthly rolling measure of the year up to, and including, the month in question, in the same way as Parry *et al*.^17^ The percentages of patients and appointments included in all measures were calculated.

It was investigated whether some patient groups had different continuity levels and/or were more likely to be included in the measurements. Patients in the top decile for number of GP appointments over the 3 years from April 2022 until March 2025 were compared with all other patients with appointments. Patients aged ≥65 years (at March 2025) were compared with those aged <65 years. Using the electronic Frailty Index (eFI)^18^ at March 2025, patients in the severe or moderate frailty range were compared with those with mild or no frailty. As under-18s have no frailty score, patients aged <18 years at March 2025 were compared with all adult patients (that is, those aged ≥18 years).

Simple linear regression analysis was used to compare the mSLICC and SLICC. Measures were calculated using Excel (version 2508) and statistical analyses were carried out in Excel or Stats Direct 2.6.5.

## Results

### Population and consultations included

Practice A provided 17 312 GP appointments to 5369 different patients from April 2024 until March 2025 (Table 1) and practice B provided 11 815 GP appointments to 4555 different patients (Table 2). The SLICC included all appointments; the mSLICC included:

- 73.3% of appointments and a mean of 71.2% (standard deviation [SD] 2.2%) of patients with appointments each month in practice A;
- 60.5% of appointments and a mean of 59.1% (SD 1.2%) of patients with appointments in practice B; and
- 68.1% (19 840 of 29 127) of appointments and a mean of 65.2% (SD 6.4%) of patients with appointments for both practices combined.

Patients could be excluded from the mSLICC either because they had fewer than two appointments in the previous 2 years or because they had not seen any individual GP more than once. Of the patients not included in the mSLICC in the example month (March 2025), this was due to having fewer than two total appointments in the previous 2 years for 250 of 376 (66.5%) patients in practice A (Supplementary Figure S1), and 184 of 393 (46.8%) patients in practice B (Supplementary Figure S2).

The UPC and BB for April 2024–March 2025 included 44.5% of patients but 75.9% of appointments in practice A (Table 1), and 37.5% of patients but 66.8% of appointments in practice B (Table 2). In the two combined, this was 21 032 (72.2%) out of 29 127 appointments and 4096 of 9924 (41.3%) patients.

**Table 1.** Practice A: numbers and percentages of appointments and patients included in the various continuity measures for April 2024–March 2025^a^

Group on 31 March 2025	Appointments							Patients with appointments					
	Total, *n*	Included in mSLICC			Included in UPC and BB			Total	Included in mSLICC		Included in UPC and BB		
		*n*	%	*P*-value, χ^2^	*n*	%	*P*-value, χ^2^		Mean %	*P*-value, *t*-test	*n*	%	*P*-value, χ^2^
Whole population	17 312	12 695	73.3		13137	75.9		5369	71.2		2388	44.5	
Top decile of GP appointment numbers over 3 years	6133	6098	99.4	<0.001	5961	97.2	<0.001	834	99.6	<0.001	727	87.2	<0.001
Lowest nine deciles of GP appointment numbers over 3 years	11 179	6597	59.0		7176	64.2		4535	58.9		1661	36.6	
Aged ≥65 years	5726	4912	85.8	<0.001	4942	86.3	<0.001	1329	84.3	<0.001	776	58.4	<0.001
Aged <65 years	11 586	7783	67.2		8195	70.7		4040	65.4		1612	39.9	
Male	6921	4782	69.1	<0.001	4980	72.0	<0.001	2331	66.9	<0.001	930	39.9	<0.001
Female	10 391	7913	76.2		8157	78.5		3038	74.1		1458	48.0	
Severe or moderate frailty (eFI)	3027	2881	95.2	<0.001	2856	94.4	<0.001	474	94.4	<0.001	359	75.7	<0.001
Mild or non-frail (eFI)	12 576	8919	70.9		9195	73.1		4208	69.3		1808	43.0	
Aged <18 years	1709	895	52.4	<0.001^b^	1086	63.5	<0.001^b^	687	50.4	<0.001^b^	221	32.2	<0.001^b^

^a^The number of patients included in the mSLICC is not given as some patients may be included in some months (when they have a regular GP) and not others; the mean of the percentage of all patients with appointments each month is given instead. The SLICC includes all appointments. ^b^Compared with whole adult population. BB = Bice–Boxerman Continuity of Care Index. eFI = electronic Frailty Index. mSLICC = modified St Leonard’s Index of Continuity of Care. UPC = Usual Provider of Care Index.

Table 1 (practice A) and Table 2 (practice B) show that patients who were frequent attenders over the 3 years, aged ≥65 years, female, or in the moderate or severely frail categories on the eFI, were all more likely to be included, and have their appointments included, in all four measures. These differences reflect that all these groups have higher numbers of appointments. Patients aged <18 years were less likely to be included in all measures.

**Table 2.** Practice B: numbers and percentages of appointments and patients included in the various continuity measures for April 2024–March 2025^a^

Group on 31 March 2025	Appointments							Patients with appointments					
	Total, *n*	Included in mSLICC			Included in UPC and BB			Total	Included in mSLICC		Included in UPC and BB		
		*n*	%	*P*-value, χ^2^	*n*	%	*P*-value, χ^2^		Mean, %	*P*-value, *t*-test	*n*	%	*P*-value, χ^2^
Whole population	11 815	7145	60.5		7895	66.8		4555	59.1		1708	37.5	
Top decile of GP appointment numbers over 3 years	3097	3037	98.1	<0.001	2991	96.6	<0.001	522	98.3	<0.001	458	87.7	<0.001
Lowest nine deciles of GP appointment numbers over 3 years	8718	4108	47.1		4904	56.3		4033	47.3		1250	31.0	
Aged ≥65 years	3103	2307	74.3	<0.001	2299	74.1	<0.001	1062	73.7	<0.001	503	47.4	<0.001
Aged <65 years	8712	4838	55.5		5596	64.2		3493	53.8		1205	34.5	
Male	4685	2501	53.4	<0.001	2899	61.9	<0.001	1972	52.2	<0.001	648	32.9	<0.001
Female	7130	4644	65.1		4996	70.1		2583	63.7		1060	41.0	
Severe or moderate frailty (eFI)	831	722	86.9	<0.001	739	88.9	<0.001	186	85.9	<0.001	124	66.7	<0.001
Mild or non-frail (eFI)	9300	5647	60.7		6079	65.4		3673	59.4		1344	36.6	
Aged <18 years	1684	776	46.1	<0.001^b^	1077	64.0	<0.001^b^	696	44.8	<0.001^b^	240	34.5	<0.001^b^

^a^The number of patients included in the mSLICC is not given as some patients may be included in some months (when they have a regular GP) and not others. The mean of the percentage of all patients with appointments each month is given instead. The SLICC includes all appointments. ^b^Compared with whole adult population. BB = Bice–Boxerman Continuity of Care Index. eFI = electronic Frailty Index. mSLICC = modified St Leonard’s Index of Continuity of Care. UPC = Usual Provider of Care Index.

### Continuity measures

Continuity scores for practice A are outlined in Table 3: the overall mSLICC score (percentage of appointments with the patients’ regular GP) was 52.0%, the SLICC score (percentage of appointments with the named GP) was 57.1%, the mean patient UPC score for that year was 0.63 (95% CI = 0.62 to 0.64), and the mean patient BB score was 0.41 (95% CI = 0.40 to 0.43). Scores for practice B are shown in Table 4: the mSLICC score was 25.9%, the SLICC score was 25.3%, the mean patient UPC score for the year was 0.50 (95% CI = 0.49 to 0.51), and the mean BB score was 0.23 (95% CI = 0.22 to 0.25). The monthly results for the mSLICC and SLICC, and the mean rolling 1-year UPC and BB scores (up to, and including, the month) are shown in Figure 1. Across both practices, the mSLICC correlated with the SLICC (r = 0.98, *P*<0.001).

**Table 3.** Practice A: continuity measures for the whole practice population and comparisons between patient groups

Group on 31 March 2025	mSLICC		SLICC		UPC		BB	
	%	*P*-value, χ^2^	%	*P*-value, χ^2^	Mean	*P*-value, *t*-test	Mean score	*P*-value, *t*-test
Whole population	52.0		57.1		0.63		0.41	
Top decile of GP appointment numbers over 3 years	51.8	0.70	56.3	0.15	0.61	0.005^a^	0.41	0.44
Lowest nine deciles of GP appointment numbers over 3 years	52.1		57.5		0.64		0.42	
Aged ≥65 years	54.9	<0.001^a^	58.6	0.003^a^	0.64	0.37	0.43	0.13
Aged <65 years	50.1		56.3		0.63		0.41	
Male	53.4	0.01^a^	58.3	0.008^a^	0.65	0.009^a^	0.43	0.02^a^
Female	51.1		56.3		0.62		0.40	
Severe or moderate frailty (eFI)	53.0	0.61	56.9	0.04^a^	0.62	0.03^a^	0.41	0.17
Mild or non-frail (eFI)	53.6		58.9		0.65		0.43	
Aged <18 years	32.4	<0.001^a,b^	43.4	<0.001^a,b^	0.51	<0.001^a,b^	0.26	<0.001^a,b^

^a^Statistically significant. ^b^Compared with whole adult population. BB = Bice–Boxerman Continuity of Care Index. eFI = electronic Frailty Index. mSLICC = modified St Leonard’s Index of Continuity of Care. SLICC = St Leonard’s Index of Continuity of Care. UPC = Usual Provider of Care Index.

**Table 4.** Practice B: continuity measures for the whole practice population and comparisons between patient groups

Group on 31 March 2025	mSLICC		SLICC		UPC		BB	
	%	*P*-value, χ^2^	%	*P*-value, χ^2^	Mean	*P*-value, *t*-test	Mean	*P*-value, *t*-test
Whole population	25.9		25.3		0.50		0.23	
Top decile of GP appointment numbers over 3 years	26.2	0.58	26.9	0.02^a^	0.48	0.003^a^	0.24	0.76
Lowest nine deciles of GP appointment numbers over 3 years	25.6		24.7		0.51		0.23	
Aged ≥65 years	32.9	<0.001^a^	33.6	<0.001^a^	0.55	<0.001^a^	0.29	<0.001^a^
Aged <65 years	22.5		22.3		0.48		0.21	
Male	29.9	<0.001^a^	27.1	<0.001^a^	0.52	<0.001^a^	0.27	<0.001^a^
Female	23.7		24.1		0.49		0.21	
Severe or moderate frailty (eFI)	31.3	0.013^a^	32.4	<0.001^a^	0.52	0.85	0.26	0.50
Mild or non-frail (eFI)	26.9		26.0		0.51		0.25	
Aged <18 years	13.1	<0.001^a,b^	17.8	0.001^a,b^	0.42	<0.001^a,b^	0.14	<0.001^a,b^

^a^Statistically significant. ^b^Compared with whole adult population. BB = Bice–Boxerman Continuity of Care Index. eFI = electronic Frailty Index. mSLICC = modified St Leonard’s Index of Continuity of Care. SLICC = St Leonard’s Index of Continuity of Care. UPC = Usual Provider of Care Index.

**Figure 1.**
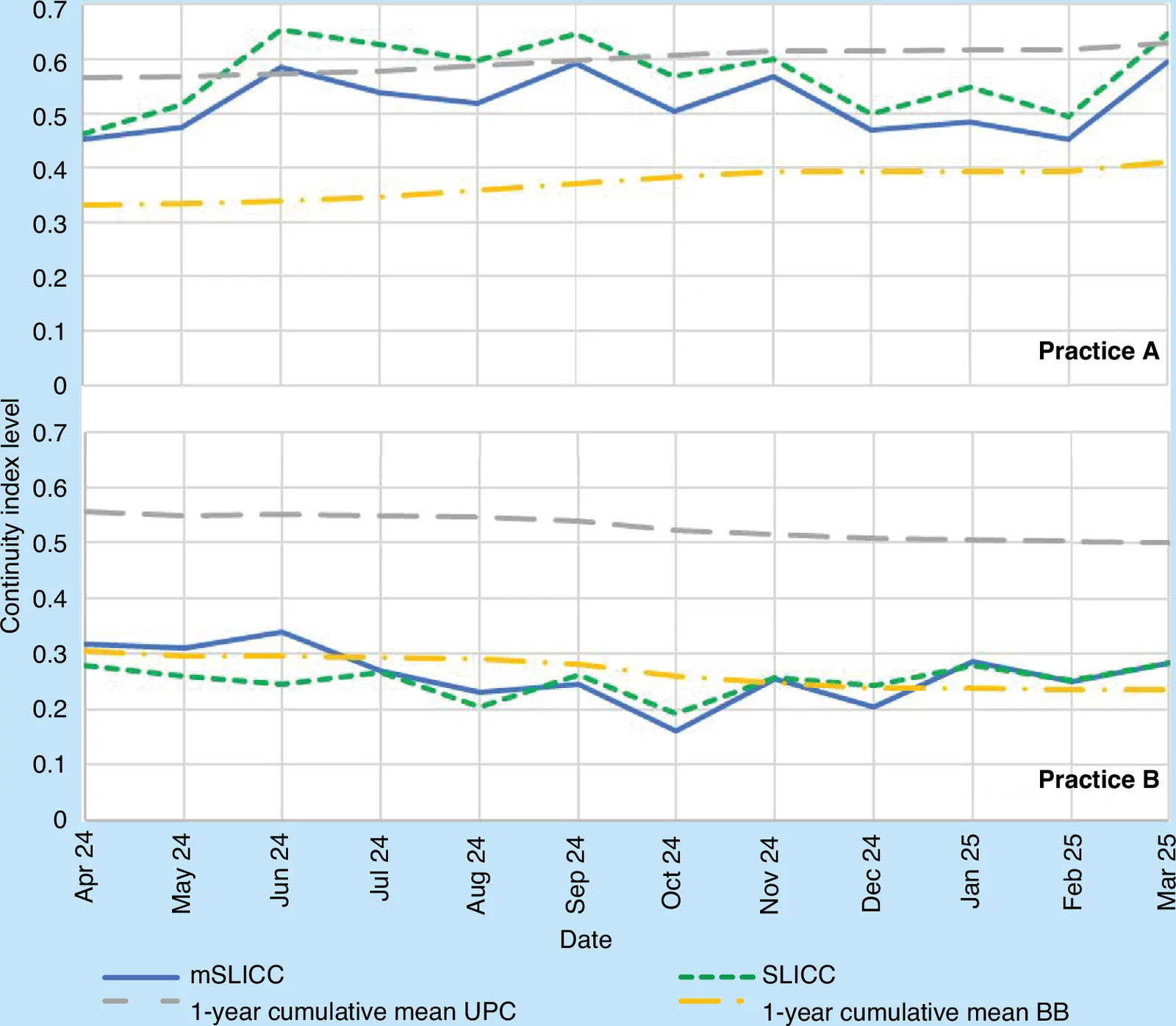
Results for each practice for 1 year (April 2024–March 2025) for SLICC (monthly), mSLICC (monthly), UPC (rolling cumulative 1 year^a^), and BB (rolling cumulative 1 year^a^). ^a^Takes in the 12 months up to, and including, the month named. BB = Bice–Boxerman Continuity of Care Index. mSLICC = modified St Leonard’s Index of Continuity of Care. SLICC = St Leonard’s Index of Continuity of Care. UPC = Usual Provider of Care Index.

Tables 3 and 4 show, for practice A and practice B respectively, the continuity levels assessed by different measures when patients were grouped by age, sex, frailty category, and total appointment numbers over 3 years. Patients who were aged <18 years or female had less continuity, compared with adults or male patients, respectively, on all measures in both practices. Patients aged ≥65 years had more continuity compared with those aged <65 years in practice B on all measures, but only on the SLICC and mSLICC in practice A. In both practices, the UPC score for the top decile of attenders was lower, while other measures showed no significant difference between the top decile and the rest apart from the SLICC score for practice B which was higher for the top decile of attenders.

In practice A, the named GP was the regular GP for 82.1% (10419 out of 12695 appointments) of appointments where a regular GP could be identified when using the mSLICC; in practice B, this was 38.6% (2760 out of 7145 appointments). When using the UPC, the ‘usual’ provider was the named GP for 83.5% (1994 out of 2388 patients) of included patients in practice A and 45.0% (768 out of 1708) of included patients in practice B.

Patients not included in the mSLICC had slightly lower SLICC scores at 54.3%, compared with 58.0% (*P*<0.001), in practice A (Supplementary Table S2) and 22.8%, compared with 26.9% (*P*<0.001), in practice B (Supplementary Table S3). However, for frequent attenders, patients aged ≥65 years, more frail patients and patients aged <18 years, the SLICC scores were not statistically significantly lower for patients not included in the mSLICC (Supplementary Tables S2 and S3).

## Discussion

### Summary

The mSLICC is a new method of continuity measurement designed to be applicable in practices with, and without, defined personal lists. Unlike the UPC or BB, which have a minimum appointment requirement in the timeframe measured, the mSLICC included a substantial proportion of patients and, as such, a greater number of less-frequent attenders. As monthly measurement was possible, the tool was reactive to changes in the practice. The difference between the SLICC and the mSLICC is the use of the regular GP, rather than the named, personal-list-holding GP, meaning not all patients could be included in the mSLICC as some had no regular GP.

The SLICC and mSLICC would be expected to differ most in practices where continuity is provided by GPs who are not the official named GP, as these practices would have more appointments for which the previously identified regular GP is not the named GP. However, the mSLICC correlated strongly with the SLICC in the two practices in this study.

### Strengths and limitations

The use of the mSLICC was demonstrated in two practices only and, as these are from the same primary care network, there is limited variation between them in terms of setting and patient population.

By identifying a regular GP, then asking the binary question of whether each appointment in a month is with that patient’s regular GP, the mSLICC can be used to measure continuity, on a monthly basis, in practices that do, and do not, have personal lists. This dynamic measurement allows for closer monitoring of the impact of staff changes, service redesign, or network-level developments, all of which may positively or negatively affect continuity. In practices with personal lists, the mSLICC is likely to reflect the continuity provided by named GPs, as measured using the SLICC. However, the continuity measured by the mSLICC could be considered more representative of real-world continuity because having three appointments in 2 years and 1 month with one GP is more likely to reflect actual familiarity than the SLICC, which will include patients who have not previously seen their named GP.

The mSLICC does not capture continuity with more than one clinician and, as such, will not measure micro-team continuity without further adaptation. It is also designed to capture therapeutic and episodic continuity, and not the distributed work of coordination with other team members and secondary care.^19^ As a previously named GP is not required, mSLICC calculations could include other members of the multidisciplinary team; however, this was not done as there is little evidence of benefit for continuity with other clinicians. Not all patients could be included in the mSLICC, so some appointments and patients were excluded; most of these — including newly registered patients and infrequent attenders — had insufficient prior appointments to acquire a regular GP. Patients with no existing continuity were also be excluded. It was found that patients excluded from the mSLICC were, at a whole-population level, less likely to have seen their named GP (so had lower SLICC scores), but this was not the case if they were in some of the groups thought most likely to benefit from continuity: frequent attenders, older people, more frail people, or children (Supplementary Figures S2 and S3).

GP turnover may cause the mSLICC score to decrease for a while, as patients transition from one regular GP to another. A major limitation of the mSLICC is that it requires 2 years and 1 month’s worth of data; this means it will not be workable in practices with recent clinical system changes as data would be unavailable. The mSLICC is also more difficult to calculate than the SLICC, as identifying the regular GP is complex and would need to be redone each month. Specific software will be needed to enable most practices to use the mSLICC. An extension of this could be regular-GP-specific continuity measurements and a personal-list-building tool.

### Comparison with existing literature

Several measures for continuity have been published and used in research.^9,10,20–22^ Continuity as measured using these has been shown to be associated with a range of positive outcomes for patients.^1,3,4^ The UPC and BB levels found in practice B were close to national levels from 2019–2021^17^ (mean UPC scores 0.50 to 0.53; BB 0.27 to 0.29) also using a minimum of three appointments over 1 year. Practice A scored higher than these national levels.

However, there are problems with the UPC and BB measures. With two appointments (as in numerous studies), two possible values for the UPC exist: 0.5 (no continuity) or 1 (meaning two appointments with one GP). Patients with two appointments and no continuity skew the UPC upwards; this is particularly problematic as these patients are counted equally to the 0.5 score of a patient with five out of 10 appointments with the same GP, which arguably is continuity. Even with three appointments as the minimum, the UPC score is 0.33 without continuity. The BB may appear low when GPs provide good, but not perfect, continuity. For both measures, an appointment with a frequent attender contributes less to the mean than one with someone with the minimum for inclusion. These biases explain the lower UPC results for frequent attenders.

The SLICC^7^ was specifically developed for measurement within UK general practice. It allows monthly measurement and includes all GP appointments, while counting each appointment equally. The drawback is the requirement for a pre-existing named GP with whom the patient is expected to have continuity. The mSLICC overcomes this. The method used for the mSLICC for identifying the regular GP approximates the method used by Kajaria-Montag *et al*,^23,24^ who found that seeing the regular GP was associated with a longer GP reconsultation interval plus reduced emergency department use and hospital admissions.

### Implications for research and practice

The mSLICC could be an effective health-improvement tool to enable practices with, or without, personal lists to measure and improve continuity. It could be scaled up to regional or national level as, like the SLICC, it counts each appointment equally and allows for monthly measurements. For those wishing to track continuity improvement at scale, the mSLICC overcomes a major disadvantage of using the UPC and BB, which is that, by focusing on less-frequent attenders, practices could potentially manipulate their UPC or BB scores without increasing overall continuity. These existing measures also could be biased against practices with high demand and attendance rates.

Improved, integrated IT systems are needed to allow for the wider adoption of the mSLICC and more dynamic processing. In addition, further evaluation should be conducted with a larger number of practices and, potentially, other members of the clinical team. The mSLICC provides a method to measure continuity of care within a short timeframe, in all practices, with good statistical and face validity.
